# Mortality due to low-quality health systems in the universal health coverage era: a systematic analysis of amenable deaths in 137 countries

**DOI:** 10.1016/S0140-6736(18)31668-4

**Published:** 2018-11-17

**Authors:** Margaret E Kruk, Anna D Gage, Naima T Joseph, Goodarz Danaei, Sebastián García-Saisó, Joshua A Salomon

**Affiliations:** a Department of Global Health and Population, Harvard T H Chan School of Public Health, Boston, MA, USA; b Department of Gynecology and Obstetrics, Emory University School of Medicine, Atlanta, GA, USA; c Ministry of Health, Mexico City, Mexico; d Center for Health Policy and Center for Primary Care and Outcomes Research, Stanford University School of Medicine, Stanford, CA, USA

## Abstract

**Background:**

Universal health coverage has been proposed as a strategy to improve health in low-income and middle-income countries (LMICs). However, this is contingent on the provision of good-quality health care. We estimate the excess mortality for conditions targeted in the Sustainable Development Goals (SDG) that are amenable to health care and the portion of this excess mortality due to poor-quality care in 137 LMICs, in which excess mortality refers to deaths that could have been averted in settings with strong health systems.

**Methods:**

Using data from the 2016 Global Burden of Disease study, we calculated mortality amenable to personal health care for 61 SDG conditions by comparing case fatality between each LMIC with corresponding numbers from 23 high-income reference countries with strong health systems. We used data on health-care utilisation from population surveys to separately estimate the portion of amenable mortality attributable to non-utilisation of health care versus that attributable to receipt of poor-quality care.

**Findings:**

15·6 million excess deaths from 61 conditions occurred in LMICs in 2016. After excluding deaths that could be prevented through public health measures, 8·6 million excess deaths were amenable to health care of which 5·0 million were estimated to be due to receipt of poor-quality care and 3·6 million were due to non-utilisation of health care. Poor quality of health care was a major driver of excess mortality across conditions, from cardiovascular disease and injuries to neonatal and communicable disorders.

**Interpretation:**

Universal health coverage for SDG conditions could avert 8·6 million deaths per year but only if expansion of service coverage is accompanied by investments into high-quality health systems.

**Funding:**

Bill & Melinda Gates Foundation.

## Introduction

Universal health coverage (UHC) has been embraced by global organisations such as WHO and the World Bank as a means to improve health and reduce the financial burden from receiving care. UHC is a central plank of the Sustainable Development Goals (SDGs), the ambitious new development targets that were signed by 193 UN member states to improve health and development by 2030.^1^ Although financing and implementation of UHC will differ by country, the common definition is the ability of all people to obtain good-quality services when they need them without facing financial hardship.^2^

Supporters of UHC have promoted it as a means for improving population health.^3^ These supporters theorise that expanding health insurance would promote the utilisation of health services that reduce mortality and morbidity. However, although insurance generally increases use of services, evidence on mortality reductions is mixed. Escobar and colleagues^4^ found that health insurance was associated with improved health status in only three of nine studies in low-income and middle-income countries (LMICs; from Vietnam, China, and Brazil). In the USA, coverage has been associated with better self-reported health status and in one recent study,^5^ with reduced mortality. The lack of consistent evidence on health benefits from insurance coverage might be in part due to methodological challenges because mortality is multifactorial and subject to factors outside of health care; people purchasing insurance are more unwell on average, and deaths are relatively rare and require large studies to measure their prevalence. However, insurance expansion might also be unsuccessful in improving outcomes if no effective treatment is available for a given condition or if quality of care is poor.

In low-income countries, evidence is emerging that expanding health care coverage does not necessarily result in better outcomes, even for conditions highly amenable to medical care. A large programme called Janani Suraksha Yojana, that was set up 13 years ago in India, has provided cash incentives for women to deliver their children in health facilities and has increased coverage of facility birth for more than 50 million women, but these incentives have not improved maternal or newborn survival.^6^, ^7^ Many of the births in this programme occurred in primary care centres that did not have sufficiently skilled staff to address maternal and newborn complications.^8^ Similarly, low quality of care for mothers and children has been documented in primary care facilities in Africa and in India.^9^, ^10^, ^11^ Researchers have also found large deficiencies in quality of hospital care for surgical conditions, obstetric care, and care of tuberculosis,^12^, ^13^, ^14^ whereas other studies^15^ have shown large differences between treatment and successful control of blood pressure.^15^

**Research in context**

**Evidence before this study**

Although amenable mortality has been estimated and discussed in high-income countries for several decades, the concept has only recently been extended to low-income countries. We searched PubMed for the terms “amenable mortality” and “quality” for studies published in English from 1990–2018 and reviewed citations in relevant articles. Nolte and McKee have developed the concept of amenable mortality to estimate the number of deaths that could be averted by health care in Organisation for Economic Co-operation and Development countries. In 2016, the Global Burden of Disease (GBD) group extended this concept to low-income and middle-income countries (LMICs) and developed an access and quality index to compare performance. Multicountry studies, such as those by Souza and colleagues and Biccard and colleagues, have shown that in some LMICs mortality is higher for people receiving care in facilities than in high-income countries, even after adjusting for morbidity. Alkire and colleagues found that worldwide 8 million deaths were amenable to health care, resulting in estimated welfare losses of US$6·0 trillion to LMICs in 2015.

**Added value of this study**

This study reports the number of deaths amenable to health care in LMICs and is the first to estimate the proportion of these deaths due to poor quality of care versus non-utilisation of care. This finding has important policy implications for countries pursuing universal health coverage as increased access to poor quality of care is unlikely to improve health outcomes. Our study found that nearly 8 million people die every year because of a lack of access to high-quality care. We found a higher proportion of amenable deaths are among health system users than non-users in LMICs. Deaths caused by poor-quality health care spanned the conditions included in the Sustainable Development Goals, including cardiovascular diseases, neonatal conditions and road traffic accidents. Although the 2016 GBD study did not report numbers of amenable deaths or partition these deaths into the separate contributions of quality of care and utilisation, it did observe substantial disparities in amenable mortality across regions and related to levels of development.

**Implications of all the available evidence**

Although our findings cannot be directly compared to the study by Nolte and McKee because the conditions they reported were different in high-income settings, the authors made different adjustments for public health interventions, and the settings of care were much better resourced than in many countries in our study, and they found that mortality in 21% of men and 30% of women under the age of 75 years is amenable to good health care; the corresponding figure from our study is 56% (all amenable deaths/avertable deaths).

The 2016 GBD paper concluded that despite progress since 1990, improved access to care and quality of care received has a large potential for improving outcomes in low-income and middle-income countries, although there is a large and growing heterogeneity of performance across countries. Specifically, although many countries lag behind peers in their income group, some middle-income countries with recent health system reforms appear to be realising outsized health gains. Our estimate that 55% of all avertable mortality for Sustainable Development Goal conditions can be addressed by good health care is somewhat higher than the Nolte and McKee study estimates and suggests that health systems are just as crucial for overall mortality reduction in lower-income countries as they are in high-income countries. Our paper uniquely estimates the portion of amenable mortality due to non-utilisation of available care versus utilisation of poor quality of care. We conclude that access is no longer the only binding constraint for improving survival in LMICs—health system quality must be improved simultaneously. This is particularly trenchant as countries embark on universal health coverage, which has been characterised in terms of improved coverage and reduced financial risk. Our work, in combination with past research, shows that improving health system quality is an immediate priority if countries hope to succeed in reaching the third Sustainable Development Goal.

The evidence of poor-quality health care challenges the assumption that increasing utilisation of health services will be sufficient to reduce mortality in lower-income countries. However, to date, there have not been any studies quantifying the potential role of better-quality services versus greater coverage in reducing mortality for conditions amenable to medical care. This report will estimate the excess deaths amenable to health care in LMICs and the relative contributions of non-utilisation of health-care services and receipt of poor-quality care to these deaths.

## Methods

### Overview

Broadly, we estimated excess mortality for SDG conditions amenable to health care, after excluding deaths that could be prevented through public health and other interventions outside the health system. To estimate amenable mortality in LMICs, we compared mortality by age and sex groups in each country with corresponding mortality from a reference group of 23 high-income countries with strong universal health coverage and good health outcomes (appendix p 17). We then apportioned amenable mortality into two components: deaths due to poor quality of care (in those who used health-care services) and those due to non-utilisation of health care.

### Parameter selection

We first identified conditions for which personal health care plays an important role in reducing mortality. We began with the list of conditions identified by Nolte and McKee^16^ as amenable to health care, and further included conditions in SDG Target 3 (improved health)^17^ for which risk of death can be reduced by use of personal health care and does not require advanced technology, resulting in identification of 61 conditions in total. Because low-income countries might not have the resources to guarantee care for all 61 conditions, we also analysed a more limited subset of 41 highest priority conditions that require relatively basic interventions (appendix p 3). We applied established age ranges for which the health system could reasonably avert deaths from each condition.^18^, ^19^, ^20^ Table 1 lists the 61 included conditions and age ranges.

**Table 1:** Conditions amenable to health care

		**Age range**
HIV or AIDS		0–74
Tuberculosis		0–74
Vaccine preventable diseases		
	Hepatitis B	0–74
	Meningitis	0–14
	Diphtheria	0–14
	Otitis media	0–74
	Varicella and herpes zoster	0–74
	Whooping cough	0–4
	Meningococcal meningitis	0–14
	Measles	0–14
	Tetanus	0–74
Neglected tropical diseases		
	Cystic echinococcosis	0–74
	Cysticercosis	0–74
	Schistosomiasis	0–74
	Yellow fever	0–74
	African trypanosomiasis	0–74
	Intestinal nematode infections	0–74
	Chagas disease	0–74
	Leishmaniasis	0–74
	Dengue	0–74
	Encephalitis	0–74
Other infectious diseases		
	Malaria	0–74
	Intestinal infectious diseases	0–74
	Diarrhoeal disease	0–49
	Upper respiratory infections	0–74
	Lower respiratory infections	0–74
Maternal disorders		15–44
Neonatal disorders		0–4
Cardiovascular diseases		
	Rheumatic heart disease	0–44
	Ischaemic heart disease	0–74
	Hypertensive heart disease	0–74
	Ischaemic stroke	0–74
	Intracerebral haemorrhage	0–74
	Congenital heart anomalies	0–14
	Chronic kidney disease due to hypertension	0–49
Gastrointestinal disorders		
	Peptic ulcer disease	0–74
	Appendicitis	0–74
	Inguinal and femoral hernia	0–74
	Gallbladder and biliary diseases	0–74
	Paralytic ileus and intestinal obstruction	0–74
Diabetes		
	Diabetes mellitus	0–49
	Chronic kidney disease due to diabetes	0–49
Cancers		
	Breast cancer	0–74
	Cervical cancer	15–44
	Colon and rectum cancer	0–74
	Uterine cancer	0–74
	Malignant skin melanoma	0–74
	Non-melanoma skin cancer	0–74
	Testicular cancer	0–74
	Thyroid cancer	0–74
	Hodgkin's lymphoma	0–74
	Leukaemia	0–74
Chronic respiratory diseases		
	Asthma	0–14
	Chronic obstructive pulmonary disorder	0–74
Neurological and mental health disorders		
	Epilepsy	0–74
	Self-harm	10–74
	Alcohol use disorders	15–74
	Drug use disorders	15–74
Road injuries		0–74
Exogeneous causes		
	Poisonings	0–74
	Adverse effects of medical treatment	0–74

In assigning amenable deaths to poor quality versus non-utilisation, we assumed that once users seek care in the health system, correct management and retention in care is the system's responsibility. Retention in care (ie, repeat utilisation) is a frequently used measure of health system quality for conditions that require a course of continuous care, such as HIV and non-communicable diseases as well as immunisation.^21^, ^22^, ^23^ Because condition-specific utilisation measures were not available for all 61 conditions in LMICs, we used population utilisation data for conditions with similar clinical features and level of acuity.^24^ For conditions such as neonatal HIV and vaccine preventable diseases, the health system can prevent all incidental cases when people seek preventive care. We used receipt of at least one vaccine as the utilisation measure for most vaccine preventable conditions. For conditions that arise acutely and can be treated or cured with episodic care (eg, pneumonia, appendicitis, or road injuries), we used care-seeking for the corresponding acute illness. Birth with a skilled attendant was used for maternal and newborn complications. Finally, for chronic conditions such as diabetes mellitus that should be screened for or detected when people at risk seek routine care we used health facility visit in the past year. We used condition-specific utilisation data for tuberculosis, HIV, cancer, and mental health (appendix pp 6–7).

### Data sources

Incidence, prevalence, and mortality by cause were obtained from the Global Burden of Disease study (GBD) 2016 in 5-year age groups by sex for each country.^20^, ^25^, ^26^ Population sizes were obtained from the World Bank.^27^

Health care utilisation data were obtained from household population surveys and global estimates including the World Health Surveys, Demographic and Health surveys, UNICEF Multiple Indicator Cluster Surveys, World Mental Health Surveys, and Joint United Nations Programme on HIV/AIDS and World Development Indicators databases in the most recent years available (appendix p 5). For countries for which utilisation data were not available, we imputed values on the basis of known factors that affect utilisation.^28^, ^29^, ^30^, ^31^ We regressed utilisation for each condition by gross domestic product per capita, percent of population living in rural areas, female literacy rate, land area, numbers of doctors and nurses or midwives per patient, and GBD study region on the basis of geography and epidemiology.^31^ On average, these variables explained 59% of the variation in utilisation across conditions. We then predicted missing utilisation values using a generalised linear model with a binomial link to constrain the values to between 0 and 100%. Additional details on imputation are reported in the appendix (p 8).

### Statistical analysis

Avertable mortality was defined as the sum of preventable deaths—ie, those that could be averted through public health and other population-level public health or intersectoral policies that prevent the disease or condition in the first place—and amenable deaths—ie, those deaths that could be averted by health care once a condition occurs. This distinction is approximate, as well-functioning primary care can also contribute to primary prevention (eg, by treating hypertension before it causes ischaemic heart disease). In some cases, such as vaccine-preventable diseases, health systems are the predominant means of primary prevention.

To estimate amenable mortality, we compared case fatality (CF) for LMICs against a reference case fatality (CF^ref^) from best performing countries. The CF was calculated as the cause-specific deaths divided by individuals at risk or incident or prevalent cases of that condition as applicable (appendix p 5). To reduce the influence of spurious values on our results, observations were dropped in countries that had fewer than ten deaths across all age groups by sex except those in the best performer reference group. When a CF was greater than 1 for a particular age group by sex, we replaced it with the disease's country average CF. Finally, CFs three SDs over the age mean for that sex were treated as outliers and truncated.

Countries in the best performer reference group were 23 high-income countries that scored 90 or greater on a recent UHC index that combines coverage of interventions and risk-standardised mortality for conditions amenable to personal health care (appendix p 15),^32^, ^33^ and the reference CF was computed as the average across the reference countries. For conditions for which deaths were entirely preventable within the health system, case fatality was calculated as deaths divided by total population at risk.

Amenable mortality was computed as mortality in excess of what would be expected relative to the reference case fatality level, after first accounting for preventable mortality—ie, excluding deaths among incident or prevalent cases that should have been prevented by population level interventions. We adjusted deaths for prevention by comparing incidence or prevalence in LMICs with incidence or prevalence in the reference group. The proportion of cases that could be prevented was indicated by calculating the ratio of incidence or prevalence of a given condition in the reference group compared with each country. If the incidence or prevalence ratio was less than one (ie, lower incidence in reference group than in the case group) as expected, we multiplied the ratio by the number of cases to calculate the adjusted (lower) number of cases. When the ratio was greater than 1, cases were unadjusted. Deaths due to conditions preventable within the health system rather than through public health measures (ie, neonatal HIV, vaccine-preventable conditions, and tuberculosis) were unadjusted. Formally, excess mortality in LMICs amenable to personal health care was calculated as follows:

$$Mortality_{ijk}^{amenable}=CF_{ijk}\times Cases_{ijk}^{adj}-CF_{ijk}^{ref}\times Cases_{ijk}^{adj}$$

for age group *i*, sex *j*, and country *k*, where *CF* is case fatality, *Cases*^adj^ is the cases that remain after excluding those that could be prevented though public health intervention, and *CF*^ref^ is the reference case fatality level.

To estimate the relative contributions of poor quality of care and non-utilisation of health care to amenable mortality, we did a second analysis that partitioned mortality into deaths among health system users and among non-users.

To estimate the counterfactual mortality that would be expected if all people who currently utilise health services received high quality care, we assumed that service users would have CF equivalent to the reference CF levels. Non-user CF is likely to be greater than the country's average CF but non-user CFs are not observed and are not directly inferable from GBD study results. Therefore, to estimate a CF for non-users we sought a plausible upper bound observed CF from the same epidemiological region (those considered poor performers). First, we computed the average CF across age group and sex groups for each cause in every country. Next, we identified the 75th percentile CF within each region for each cause. For any country with a CF below the 75th percentile, we computed a ratio of the 75th percentile CF to the CF of the country, and then multiplied this ratio by each of the country's age-group specific CFs by sex to yield the estimated CF for non-users specific to age and sex. Ratios above 3 were deemed implausible and capped, affecting 5·2% of observations. Subtracting this counterfactual from total mortality (less the portion preventable through public health intervention, as above), we derived the excess mortality among people utilising the health-care system (ie, mortality due to poor-quality services):

$$Mortality_{ijk}^{quality}=CF_{ijk}\times Cases_{ijk}^{adj}-[CF_{ijk}\times CF_{k}^{ratio}\times Cases_{ijk}^{adj}\times (1-util_{ijk})+CF_{ijk}^{ref}\times Cases_{ijk}^{adj}\times util_{ijk}]$$

for which *util* is the utilisation of services, *CF*^ratio^ is the ratio of the 75th percentile CF mortality in the region to the country's average mortality for each age group by sex, and all other variables are as defined above. Mortality caused by non-utilisation is calculated as:

$$Mortality_{ijk}^{amenable}-Mortality_{ijk}^{quality}$$

We divided poor-quality mortality by the country's total population to calculate the proportion of mortality due to poor-quality services. We multiplied the poor-quality mortality within each age group within sex by the corresponding GBD study standard life expectancy for that group to estimate years of life lost (YLL), assuming that the average age at death was at the midpoint of a given interval. We calculated preventable mortality and mortality caused by non-utilisation of services or by use of poor-quality services in LMICs by GBD region and condition type by summing across countries and age bands by sex. Uncertainty intervals were estimated by using the upper and lower bounds on the uncertainty intervals for death estimates from the 2016 GBD study. As a simplifying approximation for the aggregate uncertainty in the presence of unknown correlations between estimation errors for age groups by sex, causes, and countries, we estimated uncertainty bounds for mortality totals by treating estimation errors as fully correlated between age and sex groups for a given cause in a country, but treating errors as independent across countries and diseases.

We conducted several sensitivity analyses. As a high-income reference group might not be a feasible standard of comparison for some LMICs, we used four countries identified by the Commission on Investing in Health as best performing middle-income countries (China, Cuba, Costa Rica, and Chile) as the reference group.^34^ The incidence ratio approach to account for mortality that could be prevented by public health (ie, outside the health system) also removes deaths that can be averted through primary care (eg, proper hypertension management can reduce myocardial infarction risk). As an alternative approach, we adjusted mortality for different underlying frequencies of risk factors by applying the joint population attributable fraction of behavioural, environmental, and occupational risks for disease. This approach standardises disease risk across countries.^33^ Because the data on service utilisation from World Health Surveys were from 2002–03, we conducted a sensitivity analysis in which we increased utilisation prevalence to the next highest income group to more closely approximate current service utilisation (eg, low-income countries were given the average utilisation prevalence for lower-middle income countries). Finally, as an alternative to using so-called poor performer CFs for the mortality among non-users of the health system we assumed that non-users would simply face the country's observed prevailing CF.

All analyses were done in Stata version 14.1. We mapped mortality due to poor-quality services across LMICs using QGIS version 2.14.

### Role of the funding source

The study sponsors did not have any role in the study design, data collection, data analysis, data interpretation, or writing of the report. The corresponding author had full access to all the data in the study and had final responsibility for the decision to submit for publication. All authors reviewed the final manuscript and approved submission.

## Results

Of the 19·3 million total deaths in 2016 in LMICs from the 61 specific causes and age groups considered in this study, we estimated that there were 15·6 million avertable deaths in LMICs (95% uncertainty interval [UI] 15·4–15·9 million), including 7·0 million deaths preventable through public health intervention (UI 6·8–7·2 million), and 8·6 million amenable to health care (UI 8·5–8·8 million). The amenable deaths should be viewed as a conservative estimate because some deaths currently counted under preventable could have been averted through primary prevention in the health system. Of the excess deaths amenable to health care, an estimated 3·6 million were due to non-utilisation of health care services (UI 3·5–3·7 million), and 5·0 million were due to poor quality of available care (UI 4·9–5·2 million). 224 million YLL (UI 219–230 million) were due to poor quality of available care.

**Figure 1:**
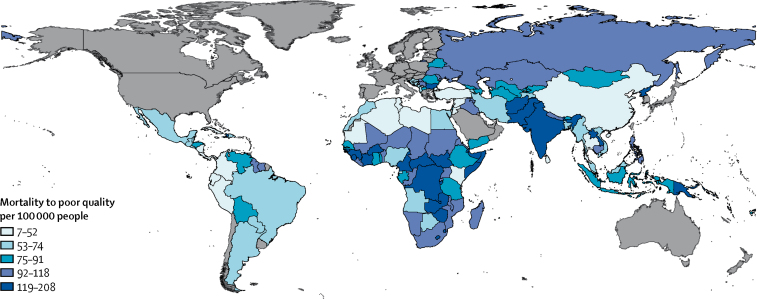
Mortality due to poor-quality health care by country

South Asia had the greatest mortality due to use of poor-quality health care at 1·9 million deaths (39% of global poor-quality service access mortality; table 2). Central Europe and Latin America had the highest percentage of amenable mortality due to receipt of poor-quality health services, whereas sub-Saharan Africa had the lowest, where a greater percentage were due to non-utilisation of services. Country results are available in the appendix (pp 9–13). Figure 1 maps mortality due to poor quality per 100 000 population. Poor-quality health care contributed to the most deaths per unit population in South Asia and central and west Africa. The total LMIC poor-quality mortality was 82 deaths per 100 000 population.

**Table 2:** Avertable and amenable mortality and mortality related to non-utilisation of services versus use of poor-quality services by region

	**Avertable deaths**		**Amenable deaths**		**Years of life lost to poor quality (per 1000 population)**
	Deaths preventable by public health interventions	Deaths amenable to health care	Deaths due to use of poor-quality services	Deaths due to non-utilisation of health services	
Andean Latin America	18 156	36 809	21 408 (58·2%)	15 401 (41·8%)	1129
Caribbean	37 167	43 742	29 861 (68·3%)	13 881 (31·7%)	1221
Central Asia	85 651	118 595	74 880 (63·1%)	43 715 (36·9%)	3322
Central Europe	41 689	53 014	41 779 (78·8%)	11 235 (21·2%)	1138
Central Latin America	40 102	208 265	143 847 (69·1%)	64 418 (30·9%)	6432
Central sub-Saharan Africa	291 999	273 717	142 044 (51·9%)	131 674 (48·1%)	8429
East Asia	875 835	1 335 030	664 893 (49·8%)	670 137 (50·2%)	23 023
Eastern Europe	428 032	294 519	187 790 (63·8%)	106 729 (36·2%)	6009
Eastern sub-Saharan Africa	804 363	721 395	349 785 (48·5%)	371 610 (51·5%)	19 668
North Africa and Middle East	440 319	521 815	325 743 (62·4%)	196 072 (37·6%)	17 590
Oceania	19 707	20 721	12 742 (61·5%)	7980 (38·5%)	559
South Asia	1 900 170	3 016 686	1 944 044 (64·4%)	1 072 641 (35·6%)	81 540
Southeast Asia	515 460	788 335	481 259 (61·0%)	307 075 (39·0%)	19 657
Southern Latin America	10 010	39 488	29 229 (74·0%)	10 258 (26·0%)	1118
Southern sub-Saharan Africa	258 889	152 119	85 709 (56·3%)	66 410 (43·7%)	4825
Tropical Latin America	78 825	210 086	157 573 (75·0%)	52 513 (25·0%)	6155
Western sub-Saharan	1 154 824	812 987	354 744 (43·6%)	458 243 (56·4%)	22 566
Total	7 001 198	8 647 323	5 047 330 (58·3%)	3 599 993 (41·6%)	224 381

Avertable mortality was defined as the sum of preventable deaths (averted through public health and other population-level intersectoral policies or interventions that prevent the disease or condition in the first place) and amenable deaths (averted by health care once a condition occurs). Amenable deaths comprised deaths due to use of poor-quality services and deaths due to non-utilisation of health services.

**Figure 2:**
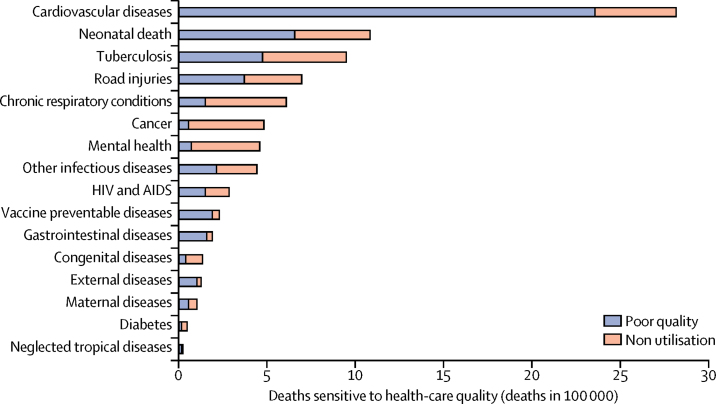
Mortality due to poor quality versus non-utilisation of health care by condition type Reproduced from Kruk and colleagues,^35^ by permission of Elsevier. External factors deaths are those due to poisonings and adverse medical events. Other infectious diseases deaths are those due to malaria, diarrhoeal diseases, intestinal infections, and upper and lower respiratory infections.

Figure 2 presents the mortality due to access to poor-quality services and non-utilisation of health services by condition type. Cardiovascular disease deaths made up 33% (2 817 000) of the amenable deaths in the total health system, of which 84% (2 358 000) were caused by use of poor-quality health services. After cardiovascular disease, deaths from neonatal conditions, tuberculosis, and road injuries comprised the most amenable deaths, with a total of 1·5 million deaths due to use of poor-quality services and 1·2 million deaths due to non-utilisation of health services. Only 11% (53 000 of 477 000) of amenable cancer deaths and 15% (69 000 of 455 000) of amenable mental and neurological deaths were due to use of poor-quality health care, reflecting the low utilisation of health services for these conditions (appendix p 14).

In our sensitivity analyses, adjusting mortality by the population attributable fraction due to environmental and behavioural risk factors to exclude deaths preventable by public health or other upstream interventions as opposed to our reference base case approach based on adjusting incidence or prevalence to reference levels in high performing countries, yielded 7·0 million amenable deaths of which 4·4 million were due to use of poor-quality health services and 2·6 million were due to non-utilisation of health services (appendix pp 3–4). Considering the full disease burden that health systems face today—ie, without subtracting deaths that were potentially preventable outside the health system—13·3 million deaths were amenable to health care, 7·6 million from use of poor-quality health care and 5·7 million from non-utilisation of any health service. Comparison with a best performing middle-income country reference group, rather than our base case reference standard from high-income countries, resulted in 3·2 million deaths due to use of poor-quality services and 2·4 million to non-utilisation of health care. Restricting the UHC package to a narrower set of conditions reduced the number of avertable deaths to 14·4 million (4·7 million due to poor-quality services and 2·8 million due to non-utilisation of services). Adjusting the World Health Surveys data on service utilisation to account for increases since 2002–03 resulted in a larger difference between non-utilisation of services (3·3 million, UI 3·2–3·4 million) and access to poor-quality services (5·3 million, UI 5·2–5·4 million). Assuming that the non-utilisation population and the population that utilised services had the same CF, we found that 5·7 million deaths were due to use of poor-quality services and 2·9 million deaths due to non-utilisation of services.

## Discussion

8·6 million people in LMICs die from causes amenable to health care; of these, 5 million are people who have used the health system but received poor-quality health care. This is five times higher than all global deaths from HIV or AIDS and over three times higher than all deaths from diabetes. Deaths attributable to receipt of poor-quality health care constitute 58% of all amenable mortality in these countries. Because deaths in LMICs occur at younger ages, poor quality of health care takes a large toll on YLL: 224 million in the study countries. The estimate of amenable mortality is conservative since some deaths currently counted under preventable could have been averted in the health system through primary prevention of the condition.

Few comparison studies are currently available. A 2017 paper by the GBD collaborators^33^ compared health systems by use of a similar approach but did not report on deaths. The authors noted large gaps between observed health system performance in many countries and the best performing comparators. Alkire and colleagues^36^ reported amenable mortality of 8 million deaths for 38 conditions in 198 countries, 96·3% of which occurred in LMICs, using somewhat different methods (reducing amenable mortality by attributable risk factors rather than differences in incidence). They estimated that this mortality would result in US$11·2 trillion in lost economic output between 2015 and 2020 in LMICs.^36^ Nolte and McKee have tracked deaths due to conditions amenable to timely and effective health care in Europe and other Organisation for Economic Co-operation and Development countries for the past 15 years.^37^, ^38^, ^39^ Our findings cannot be directly compared with their work because we adjusted incidence or prevalence for all conditions to exclude deaths that could have been prevented outside the health system, whereas Nolte and McKee only exclude a portion (50% of cardiovascular deaths). However, they found that 21% of mortality under age 75 for men and 30% for women is amenable to good-quality health care; the corresponding figure from our study is 55% (all amenable deaths of those that were avertable), suggesting that poor-quality health systems are a greater impediment to improved population health in poor than in rich countries.

Given the global focus on UHC, we designated deaths in people who presented to the health system but were not properly managed or retained in health care as deaths due to receipt of poor-quality health care and deaths in those who did not use care for each illness as deaths due to non-utilisation of services. We found that across LMICs, poor quality contributed to more deaths than non-utilisation of services, (5·0 million poor-quality health care *vs* 3·6 million non-utilisation of services). Poor quality was a larger driver of mortality than non-utilisation of services in 14 of 17 geographic regions and 115 of 137 countries, including in many of the poorest regions with high mortality. Countries at different levels of development will adopt different UHC packages that might not include all of the conditions assessed here. However, this does not lessen the importance of quality of care: our modeling shows that if low-income countries fund only less advanced care, poor-quality services will account for almost two-thirds of the amenable deaths. Multicountry studies support the finding that mortality in LMICs for people using health-care services substantially exceeds that in higher-income countries.^13^, ^40^

Poor quality was an important driver of amenable mortality across conditions, including 84% of cardiovascular mortality; 81% of vaccine preventable diseases; 61% of neonatal conditions; and half of deaths from maternal causes, road injury, tuberculosis, HIV, and other infectious diseases. Averting deaths from cancer, congenital defects, mental health, and chronic respiratory conditions will require major efforts to boost utilisation of services along with improved quality. These figures can provide insights about potential policy directions for countries. The breadth of conditions for which poor-quality health care contributes to excess mortality suggests that health system-wide improvement is needed rather than disease-specific quality interventions.

As lower-income countries undergo demographic and epidemiological transitions, they will need to implement public health measures and strengthen the quality of health systems to continue to reduce mortality. After several decades of health gains in infectious diseases and child health, the residual mortality in LMICs is comprised of more complex and multimorbid conditions. Our study provides evidence that even in settings where progress has been made on UHC, deaths due to poor-quality services represent a substantial challenge. From a financing standpoint, underperforming health systems reduce the returns on UHC investments. However, when coupled with investments in health system quality, expanding insurance can result in major health gains as shown by Thailand, Rwanda, and Costa Rica, which have pursued this dual strategy and achieved substantial improvements in survival in child and maternal health.^34^, ^41^, ^42^

Our study has several limitations. The incidence, prevalence, and mortality specific to cause, age, and sex come from the GBD, which has known limitations in estimates, particularly for causes of death in LMICs with weak or non-existent vital registration systems.^33^ Even in systems with strong vital registration systems, records can misidentify the underlying causes of death. The GBD group conducts extensive corrections to underlying data to address these. Comorbidity and disease history could be different between LMICs and high-income countries, which can result in some bias. Our approach for adjusting for primary prevention, which subtracts these deaths before the reference CF is applied, maximises preventable mortality while reducing amenable mortality. We did this to prioritise primary prevention, which is cheaper and often more effective than treatment. Some deaths that are currently categorised as preventable could have been averted through primary prevention in the health system. Our analysis does not measure morbidity so underestimates the effects of poor-quality health care on overall health. The last three points suggest that our conclusions about the health impact of poor-quality health systems are a conservative assessment.

An important limitation of our analysis is that data on health care utilisation were not complete for all conditions and countries. For conditions in which utilisation of service data were not available we used information for similar conditions and in countries where data were missing we imputed values based on factors known to affect utilisation of services. This is an imperfect approach but consistent with current literature.^43^ These data gaps mean that for some conditions, particularly those that have only recently been recognised as global health priorities, such as mental health, cancer, and road injury, our estimates of the roles of quality and utilisation of services should be considered as provisional and need to be updated when better data become available. Mortality for non-users of health services was not directly available in the data—we applied a correction to address this. However, all sensitivity analyses supported the conclusion that poor-quality health care is a larger driver of amenable mortality than utilisation of services. Finally, our characterisation of uncertainty around estimates is imperfect, given that some sources of uncertainty were not included, and aggregate level uncertainty depends on correlations between estimation errors for constituent parts, which were difficult to quantify. Collectively, these limitations call attention to the need for better data on mortality and health system use in LMICs, which will be essential for countries' efforts to track progress on UHC and other health goals.

What do the results mean for countries pursuing UHC? Each country will chart its own course on UHC, with benefit packages reflecting health priorities and available resources. However, the central role of quality is not yet sufficiently recognised in the global discourse on UHC and is underappreciated in many countries. An important starting point is better measurement of health system quality. Some countries are attempting to incorporate this into their UHC efforts. For example, South Africa has begun a nationwide Ideal Clinic programme and Tanzania has used a star-rating system to measure quality of primary care.^44^ These results are being used to identify entry points for health-system improvement. Our analysis shows that at all levels of development and across different scopes of insured services, poor quality of care will limit the mortality reduction possible from greater coverage. Countries pursing UHC must put better quality on par with expanded coverage if they are to substantially improve health.

**This online publication has been corrected. The corrected version first appeared at thelancet.com on September 20, 2018**
