# Department of Error

**DOI:** 10.1016/S0140-6736(18)32337-7

**Published:** 2018-11-17

*Kruk ME, Gage AD, Joseph NT, Danaei G, García-Saisó S, Salomon JA. Mortality due to low-quality health systems in the universal health coverage era: a systematic analysis of amenable deaths in 137 countries* Lancet *2018; https://doi.org/10.1016/S0140-6736(18)31668-4*—In figure 2 of this Article (published Online First on Sept 5, 2018), the y axis should read “deaths in 100 000s”. The affiliation for Prof Salomon should read “Center for Health Policy and Center for Primary Care and Outcomes Research, Stanford University School of Medicine, Stanford, CA, USA”. These corrections have been made to the online version as of Sept 20, 2018, and will be made to the printed Article.

