# Supplementary material for: Mortality due to low-quality health systems in the universal health coverage era: a systematic analysis of amenable deaths in 137 countries
Source: Lancet. 2018 Nov 17;392(10160):2203–12. doi: 10.1016/S0140-6736(18)31668-4 (PMC6238021; doi:10.1016/S0140-6736(18)31668-4)
Supplement: Supplementary appendix [file mmc1.pdf]

THE LANCET

Supplementary appendix

This appendix formed part of the original submission and has been peer reviewed. We post it as supplied by the authors.

Supplement to: Kruk ME, Gage AD, Joseph NT, Danaei G, García-Saisó S, Salomon JA. Mortality due to low-quality health systems in the universal health coverage era: a systematic analysis of amenable deaths in 137 countries. *Lancet* 2018; published online Sept 5. http://dx.doi.org/10.1016/http://dx.doi.org/10.1016/S0140-6736(18)31668-4.

Mortality due to low quality health systems—implications for universal health coverage

Web extra material

Table of Contents

Table S1. Sensitivity results (all updated with new data) 3

Table S2. Condition table 5

Table S3. Definitions and data sources for health utilization measures (updated data) 6

S4 Methods. Imputation for missing utilization data 8

Table S5. Country results table (updated data) 9

Table S6. Condition results 14

Figure S7. Deaths due to poor quality and non-utilization by GBD region (updated data) 16

Table S8. Guidelines for Accurate and Transparent Health Estimates Reporting (GATHER) Checklist 17

References 19

Table S1. Sensitivity results

Main results

	Estimate	Lower	Upper
Amenable	8,647,323	8,505,807	8,788,839
Poor quality	5,047,330	4,929,687	5,164,973
Non-utilization	3,599,993	3,495,958	3,704,029
Preventable	7,001,198	6,794,128	7,208,268
Avertable	15,648,521	15,363,438	15,933,604

Sensitivity 1: Alternate reference group (4 best performing LMICs)

	Estimate	Lower	Upper
Amenable	5,602,834	5,468,191	5,737,478
Poor quality	3,159,260	3,045,519	3,273,001
Non-utilization	2,443,575	2,362,485	2,524,665
Preventable	9,770,952	9,519,784	10,022,119
Avertable	12,604,032	12,330,408	12,877,656

Notes: The reference group is the four LMIC best performing countries as identified by the Commission on Investing in Health: China, Cuba, Costa Rica and Chile.(1)

Sensitivity 2: Limited UHC package

	Estimate	Lower	Upper
Amenable	7,552,747	7,416,785	7,688,710
Poor quality	4,740,948	4,628,071	4,853,825
Non-utilization	2,811,799	2,717,477	2,906,122
Preventable	6,845,469	6,638,591	7,052,348
Avertable	14,398,216	14,116,391	14,680,042

Notes: Excludes conditions which may be outside the scope of low-income UHC packages for low and low-middle income countries. 18 conditions are removed from the package: Cancers except for cervical cancer, congenital heart anomalies, poisonings, road injuries, mental and neurological disorders and chronic kidney disease. The condition list is unchanged in upper-middle income countries.

Sensitivity 3: Alternate prevention approach using JPAF

	Estimate	Lower	Upper
Amenable	6,974,740	6,796,681	7,152,799
Poor quality	4,387,670	4,230,239	4,545,102
Non-utilization	2,587,069	2,474,311	2,699,828
Preventable	4,989,560	4,894,500	5,084,620
Avertable	11,964,299	11,738,810	12,189,789

Notes: Rather than using the rate ratios, this uses the populations attributable fractions to account for preventable conditions. Population attributable fractions for environmental/occupational and behavioral risks from the Global Burden of Disease were downloaded for all conditions(2). Metabolic risks were not considered because primary health care can play a large role in these risks.(3) A joint population attributable fraction was calculated: $1 - (1 - PAF_{env/occ}) * (1 - PAF_{behavioral})$. Mediation between environmental and behavioral risk factors was not included.

Sensitivity 4: Adjusted utilization rates

	Estimate	Lower	Upper
Amenable	8,647,323	8,505,807	8,788,839
Poor quality	5,308,915	5,202,306	5,415,525
Non-utilization	3,338,408	3,243,328	3,433,488
Preventable	7,001,198	6,794,128	7,208,268
Avertable	15,648,521	15,363,438	15,933,604

Notes: Utilization rates derived from WHS – adult acute illness visit rate, visit to health facility in the past and cancer screening rate—are adjusted to account for potential increase since 2002/2003. Rates are set to the average rate of the next income group unless the utilization rate already exceeded this value. Low income countries were given the average utilization rate of low-middle income countries; low-middle income countries were given the average utilization rate of upper-middle income countries; upper-middle income countries were given the average utilization rate of high income countries.

Sensitivity 5: Assume utilizers face country's prevailing CFR

	Estimate	Lower	Upper
Amenable	8,647,323	8,505,807	8,788,839
Poor quality	5,701,497	5,612,948	5,790,047
Non-utilization	2,945,826	2,879,534	3,012,118
Preventable	7,001,198	6,794,128	7,208,268
Avertable	15,648,521	15,363,438	15,933,604

Sensitivity 6: Current health system burden

	Estimate	Lower	Upper
Amenable	13,327,479	13,067,636	13,587,322
Poor quality	7,610,659	7,409,225	7,812,094
Non-utilization	5,716,819	5,529,170	5,904,469

Notes: Does not adjust for preventable deaths

Table S2. Condition table

Conditions	Age Range	Utilization measure
Conditions for which health system prevents incident cases<br>*CFR = deaths/population of interest*		
HIV/AIDS	0-14	Skilled birth attendance
Vaccine preventable diseases (incl. Hepatitis B, Otitis Media, Varicella and herpes zoster, and Tetanus)	0-74	Receipt of at least one vaccine
Vaccine preventable diseases (incl. Diphtheria, Measles, Meningococcal meningitis and Meningitis)	0-74	Receipt of at least one vaccine
Whooping cough	0-4	Receipt of at least one vaccine
Cervical cancer	15-44	Cancer screening rate
Tuberculosis	0-74	Case detection rate
Acute conditions that are treated or cured with episodic care<br>*CFR= deaths/incidence*		
Neglected tropical diseases (incl. Cystic echinococcosis, cysticercosis, shistosomiasis, yellow fever, African trypanosomiasis, intestinal nematode infections, Chagas disease, leishmaniasis, dengue, encephalitis)	0-74	Infectious illness and child acute illness utilization
Other infectious diseases (incl. malaria, intestinal infectious diseases, upper and lower respiratory infections)	0-74	Infectious illness and child acute illness utilization
Diarrheal disease	0-49	Infectious illness and child acute illness utilization
Tetanus	5-74	Infectious illness and child acute illness utilization
Maternal disorders	15-44	Skilled birth attendance
Neonatal disorders	0-4	Skilled birth attendance
Rheumatic heart disease	0-44	Other acute and child acute illness utilization
Ischemic heart disease	0-74	Other acute and child acute illness utilization
Ischemic stroke	0-74	Other acute and child acute illness utilization
Intracerebral hemorrhage	0-74	Other acute and child acute illness utilization
Peptic ulcer disease	0-74	Other acute and child acute illness utilization
Paralytic ileus and intestinal obstruction	0-74	Other acute and child acute illness utilization
Appendicitis	0-74	Other acute and child acute illness utilization
Inguinal and femoral hernia	0-74	Other acute and child acute illness utilization
Gallbladder and biliary diseases	0-74	Other acute and child acute illness utilization
Asthma	0-14	Other acute and child acute illness utilization
Self-harm	10-74	Depressed patients who have sought treatment in past year
Road injuries	0-74	Road accident or injury utilization
Poisonings	0-74	Utilization for acute illnesses
Adverse effects of medical treatment	0-74	N/A
Chronic conditions requiring sustained care to either cure or prevent sequelae<br>*CFR= deaths/prevalence*		
HIV/AIDS	15-74	People living with HIV who know their status
Hypertensive heart disease	0-74	Other acute and child acute illness utilization
Congenital heart anomalies	0-14	Other acute and child acute illness utilization
Diabetes mellitus	0-49	Health facility visit in past 1 year
Breast cancer	0-74	Cancer screening rate
Thyroid cancer	0-74	Cancer screening rate
Colon and rectum cancer	0-74	Cancer screening rate
Uterine cancer	0-74	Cancer screening rate
Malignant skin melanoma	0-74	Cancer screening rate
Non-melanoma skin cancer	0-74	Cancer screening rate
Testicular cancer	0-74	Cancer screening rate
Hodgkin's lymphoma	0-74	Cancer screening rate
Leukemia	0-74	Cancer screening rate
COPD	0-74	Health facility visit in past 1 year
Chronic kidney disease (due to hypertension and due to diabetes)	0-49	Health facility visit in past 1 year
Epilepsy	0-74	Depressed patients who have sought treatment in past year
Alcohol use disorders	15-74	Depressed patients who have sought treatment in past year
Drug use disorders	15-74	Depressed patients who have sought treatment in past year

Table S3. Definitions and data sources for health utilization measures

Utilization metric	Numerator	Denominator	Data sources	Conditions applied	N LMIC countries with data	Low income average[a]	Low-middle income average[a]	Upper-middle income average[a]
Infectious illness visit rate	Sought care at in- or outpatient facility	Needed health care for self in the past year for high fever, severe diarrhea or cough	World Health Survey (WHS)(4)	Malaria, intestinal infectious diseases, upper/lower respiratory infections, neglected tropical diseases	40	75%	78%	88%
Other acute illness visit rate	Sought care at in- or outpatient facility	Needed health care for self in the past year for asthma, heart disease or minor surgery	WHS	Cardiovascular disorders, digestive disorders, asthma	40	88%	88%	92%
Child acute illness visit rate	Sought care for fever, diarrhea, or cough at a health facility	Children under 5 years with fever, diarrhea or cough in the past 2 weeks	Most recent available Demographic and Health Survey (DHS)(5), Multiple Indicator Cluster Survey (MICS)(6)	Malaria, intestinal infectious diseases, upper and lower respiratory infections, neglected tropical diseases, digestive disorders, asthma, poisonings, childhood leukemia	85	46%	55%	60%
Visit to health facility in the past year	Visit with doctor, nurse or midwife in the past 12 months (outpatient)	All respondents	WHS	Diabetes mellitus, chronic obstructive pulmonary disease, chronic kidney disease	40	28%	34%	40%
Ever vaccinated	Receipt of any one dose vaccine	Children under 5 years	DHS or MICS	Hepatitis B, vaccine preventable diseases	83	90%	94%	94%
Cancer screening rate	Ever received pap smear	Women age 18-70 years	WHS	All neoplasms except childhood leukemia	40	6%	17%	38%
Road accident or injury utilization	Received medical care or treatment for injuries	In the past 12 months, involved in a road traffic accident where suffered from bodily injury or other injury that limited everyday activities due to fall, burn, poisoning, submersion in water or by a firearm, sharp weapon or act of violence	WHS	Road injury and poisonings	40	53%	58%	64%
Sought care for mental health disorder	Sought treatment in past year for depression symptoms	Depression Symptoms	World Mental Health Survey from Thornicroft et al(7)	Neurologic and mental health disorders	11	16%	22%	28%
People living with HIV who know their status	People living with HIV who know their status	HIV positive people	UNAIDS(8)	HIV over age 15	80	50%	59%	73%
TB case detection rate	Number of new and relapse TB cases	Estimate of the number of incident TB cases for	World Development Indicators (WDI) (9)	TB	137	58%	67%	79%

	diagnosed and treated in national TB control programs	the same year						
Skilled birth attendance	Births attended by skilled health staff	Total number of live births	WDI(9)	Maternal disorders, neonatal disorders, HIV under age 15	138	55%	79%	96%

[a]Includes predicted values as described in S4 Methods

S4 Methods. Imputation for missing utilization data

We imputed utilization for countries with missing data (Table S3) based on known predictors of utilization. We regressed utilization for each condition on GDP per capita, percent rural, female literacy rate, land area, doctors and nurses/midwives per capita and the GBD region. These variables explained 59% of the variation in utilization on average across conditions. We used the most recent data on the predictors available from the past fifteen years from the World Development Indicators database.(9) We excluded GBD regions from the mental health utilization prediction model due to few degrees of freedom.

We then predicted missing utilization values using a generalized linear model with a binomial link to constrain the values to between 0 and 100%. Female literacy rates were unavailable from WDI for 59 countries. Of these, 38 countries were high income and assumed to have 100% female literacy. We filled in female literacy rates for nine additional missing countries with data from the CIA World Factbook. In the remaining countries without full data for the predictors (mostly small island states), we set utilization to the GBD region mean after predicting utilization for other countries.

Table S5. Country results table

Country	Avertable deaths: Deaths preventable by population level interventions	Avertable deaths: Deaths amenable to health care	Amenable deaths: Deaths due to poor quality	Amenable deaths: Deaths due to non-utilization	Percent of amenable deaths due to poor quality	Years of life lost to poor quality (1000s)	Poor quality deaths per 100,000 people
Afghanistan	83,540	80,556	46,153	34,403	57%	2,609	142
Albania	1,222	2,407	2,081	326	86%	64	72
Algeria	17,122	28,554	17,966	10,588	63%	1,047	45
American Samoa	12	30	23	6	79%	1	23
Angola	45,581	48,092	17,075	31,017	36%	993	68
Argentina	10,010	39,488	29,229	10,258	74%	1,118	67
Armenia	1,010	2,995	1,600	1,396	53%	53	53
Azerbaijan	7,132	12,394	8,000	4,394	65%	400	82
Bangladesh	117,549	182,905	91,631	91,275	50%	3,801	57
Belarus	17,424	12,382	7,906	4,477	64%	231	83
Belize	190	281	208	73	74%	9	52
Benin	24,833	22,456	12,777	9,679	57%	786	117
Bhutan	369	864	599	265	69%	29	75
Bolivia	6,207	11,846	8,343	3,503	70%	412	78
Bosnia and Herzegovina	2,436	3,447	2,504	943	73%	64	66
Botswana	6,158	3,118	1,408	1,710	45%	62	61
Brazil	76,295	204,036	153,327	50,708	75%	5,966	74
Bulgaria	9,107	10,544	9,064	1,480	86%	240	126
Burkina Faso	62,017	38,196	21,914	16,282	57%	1,364	121
Burundi	31,054	31,476	18,355	13,120	58%	1,081	164
Cambodia	18,402	22,313	14,544	7,770	65%	651	93
Cameroon	80,697	49,751	27,895	21,855	56%	1,642	120
Cape Verde	255	384	184	200	48%	9	37
Central African Republic	22,994	19,778	10,110	9,668	51%	543	206
Chad	49,727	37,466	15,360	22,106	41%	980	110

China	847,843	1,283,099	629,765	653,334	49%	21,628	46
Colombia	4,625	33,917	22,080	11,836	65%	918	46
Comoros	700	1,111	604	507	54%	37	76
Congo	11,048	9,330	4,950	4,380	53%	299	108
Costa Rica	367	2,303	1,240	1,063	54%	51	26
Cote d'Ivoire	73,042	55,959	31,929	24,030	57%	1,733	141
Croatia	2,317	3,434	2,498	936	73%	62	59
Cuba	3,504	8,735	5,209	3,526	60%	139	46
Democratic Republic of the Congo	206,998	192,864	108,098	84,766	56%	6,484	140
Djibouti	1,500	1,718	1,134	583	66%	67	126
Dominica	28	35	30	5	85%	1	30
Dominican Republic	7,657	8,762	6,594	2,168	75%	291	63
Ecuador	3,817	11,351	6,447	4,905	57%	344	40
Egypt	104,457	93,828	63,063	30,765	67%	2,770	69
El Salvador	1,619	5,247	3,666	1,581	70%	155	60
Equatorial Guinea	2,352	1,048	482	566	46%	33	60
Eritrea	7,916	9,973	5,117	4,856	51%	232	114
Ethiopia	131,571	176,840	79,923	96,917	45%	3,601	80
Federated States of Micronesia	92	137	95	41	70%	3	95
Fiji	875	1,253	771	482	62%	30	86
Gabon	3,026	2,605	1,328	1,277	51%	77	78
Georgia	3,207	5,601	2,993	2,608	53%	114	81
Ghana	55,901	43,048	23,615	19,433	55%	1,290	86
Grenada	50	67	57	11	84%	2	57
Guatemala	7,358	16,411	10,488	5,923	64%	542	64
Guinea	39,526	30,855	16,892	13,963	55%	947	134
Guinea-Bissau	5,153	5,480	2,852	2,628	52%	147	158
Guyana	967	1,299	938	361	72%	34	117
Haiti	22,897	21,858	14,978	6,879	69%	665	140
Honduras	2,366	8,468	6,109	2,359	72%	291	75
India	1,498,027	2,438,342	1,599,870	838,473	66%	64,664	122
Indonesia	235,662	351,190	225,641	125,549	64%	9,461	88

Iran	41,077	63,044	42,546	20,499	67%	2,205	54
Iraq	43,040	49,696	36,041	13,655	73%	2,266	99
Jamaica	1,324	1,861	1,242	619	67%	54	46
Jordan	3,013	4,245	2,670	1,575	63%	179	35
Kazakhstan	16,190	27,344	20,019	7,325	73%	719	114
Kenya	87,089	52,267	19,940	32,328	38%	1,250	43
Kiribati	154	193	138	55	72%	6	138
Kyrgyzstan	4,856	7,172	4,518	2,654	63%	229	75
Laos	10,204	15,239	9,115	6,124	60%	475	134
Lebanon	1,830	1,101	399	702	36%	27	7
Lesotho	12,584	7,252	4,377	2,875	60%	235	208
Liberia	10,138	8,251	4,298	3,954	52%	246	96
Libya	3,081	3,649	2,143	1,506	59%	99	34
Macedonia	1,616	2,105	1,753	352	83%	51	83
Madagascar	50,525	50,015	25,731	24,284	51%	1,399	106
Malawi	59,542	35,363	20,234	15,128	57%	1,463	118
Malaysia	19,805	24,653	17,017	7,636	69%	543	56
Maldives	25	138	103	35	75%	3	26
Mali	58,491	37,602	18,680	18,922	50%	1,250	106
Marshall Islands	73	73	49	24	67%	2	49
Mauritania	3,916	4,879	2,108	2,771	43%	125	51
Mauritius	595	1,287	980	307	76%	33	75
Mexico	15,564	101,675	71,432	30,243	70%	3,208	56
Moldova	6,389	6,265	3,938	2,327	63%	115	109
Mongolia	3,556	3,955	2,614	1,340	66%	124	87
Montenegro	496	426	344	81	81%	9	57
Morocco	22,005	34,996	19,241	15,755	55%	866	56
Mozambique	96,122	66,515	27,315	39,200	41%	1,685	98
Myanmar	46,618	74,692	35,679	39,014	48%	1,530	66
Namibia	6,357	4,171	2,554	1,617	61%	140	102
Nepal	27,541	46,400	26,556	19,845	57%	1,114	93
Nicaragua	882	3,999	2,463	1,537	62%	114	40
Niger	74,881	44,245	22,611	21,634	51%	1,359	114

Nigeria	545,898	376,039	122,664	253,375	33%	9,062	67
North Korea	27,993	51,931	35,128	16,803	68%	1,395	139
Pakistan	256,683	348,174	225,389	122,785	65%	11,932	119
Palestine	3,156	4,722	3,652	1,070	77%	222	83
Panama	825	2,545	1,640	905	64%	72	42
Papua New Guinea	17,141	17,188	10,439	6,749	61%	468	137
Paraguay	2,530	6,050	4,245	1,805	70%	189	64
Peru	8,131	13,611	6,618	6,993	49%	373	21
Philippines	103,342	141,696	92,587	49,109	65%	3,805	92
Romania	18,564	21,729	16,764	4,965	77%	461	85
Russia	299,856	204,791	131,744	73,047	64%	4,265	91
Rwanda	16,020	17,847	11,691	6,157	66%	695	101
Saint Lucia	50	103	66	37	64%	2	33
Saint Vincent and the Grenadines	58	94	79	15	84%	3	79
Samoa	87	188	112	76	60%	3	56
Sao Tome and Principe	111	138	87	51	63%	5	43
Senegal	22,776	25,399	12,481	12,918	49%	598	83
Serbia	5,930	8,922	6,770	2,152	76%	186	95
Sierra Leone	24,094	16,601	10,549	6,052	64%	658	162
Solomon Islands	833	1,064	710	355	67%	28	118
Somalia	23,002	28,505	14,755	13,750	52%	729	137
South Africa	180,104	96,969	51,422	45,547	53%	2,864	93
South Sudan	40,536	37,470	15,562	21,908	42%	935	127
Sri Lanka	7,252	18,121	10,721	7,401	59%	319	51
Sudan	44,352	63,248	40,588	22,660	64%	2,460	101
Suriname	442	648	462	186	71%	19	92
Swaziland	5,458	3,155	1,977	1,177	63%	116	152
Syria	10,910	9,833	6,828	3,004	69%	287	37
Tajikistan	8,151	9,432	5,605	3,827	59%	329	66
Tanzania	110,802	91,921	44,613	47,309	49%	2,705	83
Thailand	31,464	49,872	21,758	28,114	44%	773	32
The Gambia	3,278	2,786	1,223	1,562	44%	64	61

Timor-Leste	869	1,481	859	622	58%	38	72
Togo	20,091	13,453	6,626	6,827	49%	302	91
Tonga	38	83	51	32	61%	2	51
Tunisia	5,300	7,266	4,052	3,213	56%	167	37
Turkey	24,030	33,489	16,526	16,962	49%	1,064	21
Turkmenistan	4,891	6,851	4,810	2,041	70%	259	89
Uganda	99,096	80,562	42,225	38,337	52%	2,553	108
Ukraine	104,362	71,081	44,202	26,879	62%	1,398	98
Uzbekistan	36,658	42,851	24,721	18,130	58%	1,095	79
Vanuatu	401	511	353	158	69%	14	118
Venezuela	6,497	33,700	24,728	8,972	73%	1,080	80
Vietnam	41,222	87,651	52,256	35,395	60%	2,025	57
Yemen	33,407	43,588	23,874	19,714	55%	1,321	89
Zambia	48,888	39,812	22,586	17,226	57%	1,237	139
Zimbabwe	48,228	37,454	23,971	13,483	64%	1,407	154

Table S6. Condition results

	Avertable deaths		Amenable deaths	
Condition	Deaths preventable by population level interventions	Deaths amenable to health care	Deaths due to poor quality	Deaths due to non-utilization
Congenital	18,289	129,452	36,203	93,248
Cardiovascular diseases	2,467,428	2,816,650	2,357,525	459,125
Other infectious	2,115,355	438,498	210,401	228,098
External	982	125,666	103,865	21,801
Neglected tropical diseases	79,848	23,838	15,697	8,141
HIV/AIDS	692,932	282,420	147,000	135,420
Cancer	10,145	477,426	52,598	424,828
Mental health	84,229	454,723	69,496	385,227
Road injuries	188,861	693,798	372,736	321,062
Gastro-intestinal diseases	143,228	184,942	153,571	31,371
Vaccine preventable	241,342	231,291	186,787	44,504
Diabetes	93,373	46,835	10,779	36,056
Neonatal	456,339	1,080,817	657,555	423,262
Tuberculosis	-	946,003	469,956	476,047
Maternal	102,984	103,908	56,634	47,274
Chronic respiratory	305,862	611,057	146,527	464,530

Table S7. Reference group: countries with score over 90 on UHC index (10)

Andorra	Italy
Australia	Japan
Austria	Luxembourg
Belgium	Netherlands
Canada	Norway
Denmark	Singapore
Finland	Slovenia
France	South Korea
Germany	Spain
Greece	Sweden
Iceland	Switzerland
Ireland	

Figure S8. Deaths due to poor quality and non-utilization by GBD region

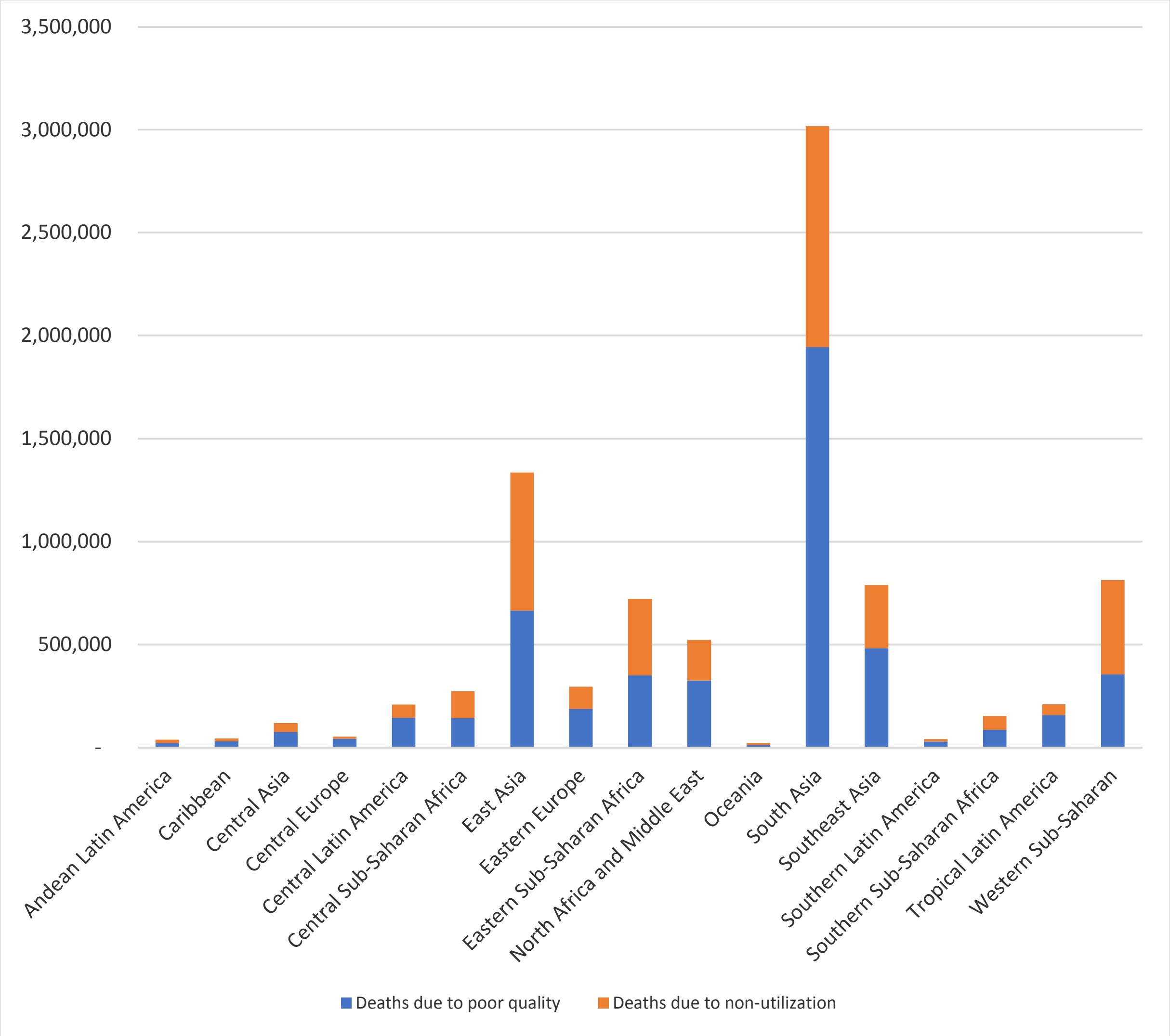

Table S9. Guidelines for Accurate and Transparent Health Estimates Reporting (GATHER) Checklist

#	GATHER checklist item	Description of compliance	Reference
**Objectives and funding**			
1	Define the indicators, populations, and time periods for which estimates were made.	Provided in main text and appendix.	Methods, Table 1, Table S2
2	List the funding sources for the work.	Funding sources listed.	Funding
**Data Inputs**			
For all data inputs from multiple sources that are synthesized as part of the study:			
3	Describe how the data were identified and how the data were accessed.	Main text includes narrative description of data collection methodology.	Methods, Table S3
4	Specify the inclusion and exclusion criteria. Identify all ad-hoc exclusions.	Main text includes narrative description of inclusion and exclusion criteria.	Methods, Table S3
5	Provide information on all included data sources and their main characteristics. For each data source used, report reference information or contact name/institution, population represented, data collection method, year(s) of data collection, sex and age range, diagnostic criteria or measurement method, and sample size, as relevant.	Main text has narrative of data downloaded from GBD; GBD online data source tool that provides metadata for data sources by component, geography, cause, and risk	Methods, Table S2; Online data tools for GBD data: http://ghdx.healthdata.org/gbd-2013-data- citations
6	Identify and describe any categories of input data that have potentially important biases (e.g., based on characteristics listed in item 5).	Description of known biases by data type included in SA.	Methods, Discussion, Table S1
For data inputs that contribute to the analysis but were not synthesized as part of the study:			
7	Describe and give sources for any other data inputs.	Appendix Table S3 lists data sources used for all utilization rates. More information on this data is available from the references.	Table S3; Appendix references
For all data inputs:			
8	Provide all data inputs in a file format from which data can be efficiently extracted (e.g., a spreadsheet as opposed to a PDF), including all relevant meta-data listed in item 5. For any data inputs that cannot be shared due to ethical or legal reasons, such as third-party ownership, provide a contact name or the name of the institution that retains the right to the data	Data inputs were obtained from publicly available online tools or published manuscripts (Higashi et al and Thornicroft et al).	Online data references:<br>GBD: http://ghdx.healthdata.org/gbd-data-tool<br>WHS:<br>http://apps.who.int/healthinfo/systems/surveydata/index.php/catalog/whs<br>DHS: https://dhsprogram.com/What-We-Do/Survey-Types/DHS.cfm<br>MICs: http://mics.unicef.org/surveys<br>AIDSinfo: http://aidsinfo.unaids.org/<br>WDI: http://wdi.worldbank.org/tables
**Data Analysis**			
9	Provide a conceptual overview of the data analysis method. A diagram may be helpful.	Main text includes description of data analysis	Methods
10	Provide a detailed description of all steps of the analysis, including mathematical formulae. This description should cover, as relevant, data cleaning, data pre-processing, data adjustments and weighting of data sources, and mathematical or statistical model(s).	Main text includes narrative description of methodology for case fatality, avertable death, quality avertable death and utilization avertable death calculations	Methods, Table S1
11	Describe how candidate models were evaluated and how the final model(s) were selected.	N/A	N/A
12	Provide the results of an evaluation of model performance, if done, as well as the results of any relevant sensitivity analysis.	Table S1 describes in detail all of the sensitivity analyses completed and the results. These are all briefly mentioned in the main text.	Table S1
13	Describe methods for calculating uncertainty of the estimates. State which sources of uncertainty were, and were not, accounted for in the uncertainty analysis.	These are described in the methods: we used the upper and lower estimates of deaths from GBD. Intervals reported in text and table	Methods, Table S1

		S1.	
14	State how analytic or statistical source code used to generate estimates can be accessed.	The main analytic do file is posted to the Harvard data repository website.	Available from https://dataverse.harvard.edu/dataset.xhtml?persistentId=doi:10.7910/DVN/DUY0UI
**Results and Discussion**			
15	Provide published estimates in a file format from which data can be efficiently extracted.	Provided in table format in main text, results section, as well as in the supplementary appendices.	Results; Supplementary files
16	Report a quantitative measure of the uncertainty of the estimates (e.g. uncertainty intervals).	Noted uncertainty estimates for main model in text of results, all other uncertainty intervals are presented in Table S1.	Results and Table S1
17	Interpret results in light of existing evidence. If updating a previous set of estimates, describe the reasons for changes in estimates.	Provided in narrative format; discussion section.	Main text: discussion section
18	Discuss limitations of the estimates. Include a discussion of any modelling assumptions or data limitations that affect interpretation of the estimates.	Provided in narrative format; discussion section.	Main text: discussion section

References

1. Jamison DT, Summers LH, Alleyne G, Arrow KJ, Berkley S, Binagwaho A, et al. Global health 2035: a world converging within a generation. Lancet. 2013;382(9908):1898–955.
2. Forouzanfar MH, Alexander L, Anderson HR, Bachman VF, Biryukov S, Brauer M, et al. Global, regional, and national comparative risk assessment of 79 behavioural, environmental and occupational, and metabolic risks or clusters of risks in 188 countries, 1990-2013: a systematic analysis for the Global Burden of Disease Study 2013. Lancet. 2015;386(10010):2287-323.
3. Oladimeji O, Access H, Quality Collaborators G. Healthcare access and quality index based on mortality from causes amenable to personal health care in 195 countries and territories, 1990-2015: a novel analysis from the Global Burden of Disease Study 2015. 2017.
4. World Health Organization. World Health Survey 2003 [Available from: http://apps.who.int/healthinfo/systems/surveydata/index.php/catalog/whs/about.
5. Demographic and Health Surveys Program. The DHS Program - Quality information to plan, monitor and improve population, health, and nutrition programs: USAID; [Available from: http://dhsprogram.com/.
6. UNICEF. Multiple Indicator Cluster Surveys (MICS) [Available from: http://mics.unicef.org/.
7. Thornicroft G, Chatterji S, Evans-Lacko S, Gruber M, Sampson N, Aguilar-Gaxiola S, et al. Undertreatment of people with major depressive disorder in 21 countries. The British journal of psychiatry : the journal of mental science. 2017;210(2):119–24.
8. UNAIDS. AIDSinfo 2016 [Available from: http://aidsinfo.unaids.org/.
9. WB. World Development Indicators. 2017.
10. Abajobir AA, Abate KH, Abbafati C, Abbas KM, Abd-Allah F, Abdulle A, et al. Measuring progress and projecting attainment on the basis of past trends of the health-related Sustainable Development Goals in 188 countries: an analysis from the Global Burden of Disease Study 2016. The Lancet. 2017;390(10100):1423-59.
11. WHO. Global Tuberculosis Report 2017. Geneva: World Health Organization; 2017.
12. World Health Organization. World malaria report 2017. Geneva: World Health Organization; 2017.
13. Watkins DA, Johnson CO, Colquhoun SM, Karthikeyan G, Beaton A, Bukhman G, et al. Global, Regional, and National Burden of Rheumatic Heart Disease, 1990–2015. New England Journal of Medicine. 2017;377(8):713-22.
14. Mogasale V, Maskery B, Ochiai RL, Lee JS, Mogasale VV, Ramani E, et al. Burden of typhoid fever in low-income and middle-income countries: a systematic, literature-based update with risk-factor adjustment. The Lancet Global health. 2014;2(10):e570-80.
15. World Health Organization. Chagas disease (American trypanosomiasis) World Health Organization,2018 [Available from: http://www.who.int/en/news-room/fact-sheets/detail/chagas-disease-(american-trypanosomiasis).
16. Status of endemicity of cutaneous leishmaniasis [Internet]. WHO. 2017 [cited June 18, 2018]. Available from: http://apps.who.int/gho/data/node.main.NTDLEISHCEND?lang=en.
17. Organization WH. Annex 1: Countries with risk of yellow fever transmission and countries requiring yellow fever vaccination. Geneva 2018.

18. Status of schistosomiasis endemic countries: 2016 [Internet]. World Health Organization. 2017 [cited June 17, 2018]. Available from: http://apps.who.int/neglected_diseases/ntddata/sch/sch.html.
19. Bhatt S, Gething PW, Brady OJ, Messina JP, Farlow AW, Moyes CL, et al. The global distribution and burden of dengue. Nature. 2013;496(7446):504.
20. Number of new reported cases (T.b. rhodesiense) Data by country [Internet]. World Health Organization. 2017. Available from: http://apps.who.int/gho/data/node.main.A1637?lang=en.
21. Number of new reported cases (T.b. gambiense) Data by country [Internet]. World Health Organization. 2017. Available from: http://apps.who.int/gho/data/node.main.A1636?lang=en.
22. WHO, cartographer Distribution of Echinococcus granulosus and cystic echinococcosis, worldwide, 2011: WHO; 2012.
23. WHO, cartographer Endemicity of Taenia solium, 2015: WHO; 2016.
24. Wang H, Liang G. Epidemiology of Japanese encephalitis: past, present, and future prospects. Therapeutics and clinical risk management. 2015;11:435-48.
25. Number of children (Pre-SAC and SAC) requiring preventive chemotherapy for soil-transmitted helminthiases: 2016 [Internet]. WHO. 2017 [cited June 18, 2018]. Available from: http://apps.who.int/neglected_diseases/ntddata/sth/sth.html.
